# Not So Innocent After All: Interfacial Chemistry Determines Charge‐Transport Efficiency in Single‐Molecule Junctions

**DOI:** 10.1002/anie.202302150

**Published:** 2023-05-04

**Authors:** Abdalghani Daaoub, James M. F. Morris, Vanessa A. Béland, Paul Demay‐Drouhard, Amaar Hussein, Simon J. Higgins, Hatef Sadeghi, Richard J. Nichols, Andrea Vezzoli, Thomas Baumgartner, Sara Sangtarash

**Affiliations:** ^1^ Device Modelling Group School of Engineering University of Warwick Coventry CV4 7AL UK; ^2^ Department of Chemistry University of Liverpool Crown Street Liverpool L69 7ZD UK; ^3^ Department of Chemistry York University 4700 Keele Street Toronto ON, M3J 1P3 Canada

**Keywords:** Charge Transport, Dithienophosphole Oxide, Interfacial Chemistry, Molecular Wires, Single-Molecule Electronics

## Abstract

Most studies in molecular electronics focus on altering the molecular wire backbone to tune the electrical properties of the whole junction. However, it is often overlooked that the chemical structure of the groups anchoring the molecule to the metallic electrodes influences the electronic structure of the whole system and, therefore, its conductance. We synthesised electron‐accepting dithienophosphole oxide derivatives and fabricated their single‐molecule junctions. We found that the anchor group has a dramatic effect on charge‐transport efficiency: in our case, electron‐deficient 4‐pyridyl contacts suppress conductance, while electron‐rich 4‐thioanisole termini promote efficient transport. Our calculations show that this is due to minute changes in charge distribution, probed at the electrode interface. Our findings provide a framework for efficient molecular junction design, especially valuable for compounds with strong electron withdrawing/donating backbones.

## Graphical abstract

The interfacial chemistry of a single‐molecule junction plays a pivotal role in determining the final charge‐transport efficiency. Internal charge reorganisation is responsible for the observed behaviour, and the results provide a general, intuitive strategy for the structural design of efficient molecular wires.

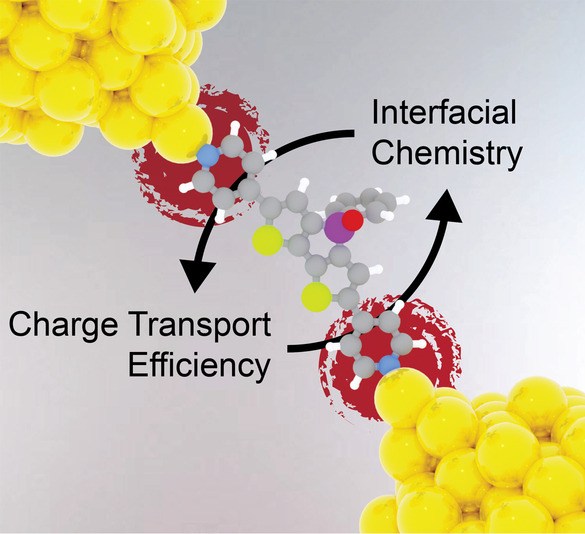

## Introduction

Real‐world application of molecular electronics depends on the ability to control charge transport through molecular junctions. To assemble a single‐molecule junction, molecules must be mechanically and electrically interfaced with two metallic electrodes, a feat generally achieved using metallophilic termini that chemisorb to the electrode surface. This interface is effectively a boundary between fully open (metallic) quantum channels and partially opened (molecular) ones, and it plays a major role in defining the overall charge‐transport efficiency by generating a “contact resistance”,[^1^, ^2^] while also determining the nature of the charge carriers (electrons or holes)^[3]^ and introducing mechanoresistive phenomena.[^4^, ^5^, ^6^] In single‐molecule junctions, however, the contacts are generally treated as resistive elements. A classic model used to rationalise charge‐transport phenomena through single‐molecule junctions based on a square energy barrier approximation^[7]^ yields the conductance G of a molecular junction expressed as $G=G_{C}e^{-\beta L}$ , where β is a tunnelling decay constant (a function of the height of the energy barrier), L is the length of the molecule, and $G_{C}$ is the *contact conductance*—a parameter describing the electronic transparency of the molecule‐electrode interface.^[8]^ This model was validated by comparison of related oligomers with different molecule/electrode interfaces showing almost identical attenuation factors β ,[^9^, ^10^, ^11^, ^12^] with charge‐transport efficiency therefore being a function of the contact conductance $G_{C}$ only. Another approximation describes the conductance of a molecular junction as $G=G_{0}T_{L}T_{M}T_{R}$ , where $G_{0}$ is the quantum of conductance (conductance of a fully open quantum channel, 2 e^2^/h≅ 77.48 μS) and $T_{L}$ , $T_{R}$ , and $T_{M}$ are, respectively, the transmission probability on the left contact, the right contact, and through the molecular backbone.[^13^, ^14^] This multiplicative case was validated by phenomena found in compounds terminated with a specific contact group translating effortlessly to similar derivatives with different chemical interfaces to the electrodes[^15^, ^16^, ^17^] and organometallic systems.[^18^, ^19^, ^20^] These concepts indeed promoted research in effective chemical interfacing to the electrodes, such as Au−C covalent bonds,[^1^, ^21^, ^22^, ^23^] chelate/multidentate anchors,[^5^, ^24^, ^25^, ^26^, ^27^] or other highly transparent molecule/electrode contacts.^[28]^

Nevertheless, segmenting the molecule into regions as described in the preceding paragraph might often not be an accurate representation of the junction, as the anchors are in intimate chemical and electronic communication with the backbone, and both influence each other. In other words, the concept that the chemical group responsible for chemically soldering the molecule to the electrodes only contributes to a “contact conductance” $G_{C}$ or a “contact transmission” $T_{L}T_{R}$ , while empirically true in many cases, has questionable real physical meaning and is unable to capture quantum phenomena such as quantum interference. The molecular structure bridging between the two anchor groups has been demonstrated to be able to change the nature of the charge carriers,^[29]^ thus demonstrating that the backbone *can* indeed influence the behaviour of the electrode contacts. Furthermore, molecular devices operate in the quantum realm and beyond the rules of conventional circuits, as evidenced by quantum interference phenomena of both constructive[^30^, ^31^, ^32^] and destructive[^33^, ^34^] nature, which have no macroscopic analogues. As some of the most widely used contacting groups, 4‐thioanisole (‐C_6_H_4_‐S‐CH_3_) and 4‐pyridyl (‐C_5_H_4_N), are electron‐rich and electron‐deficient, respectively, they could in principle respond differently to changes in the structure of the molecular backbone, thus deviating from the behaviour of simple resistive component characterised by a simple $G_{C}$ or $T_{L}T_{R}$ .

In this contribution, we shed light on the effect of the anchor group on the charge‐transport efficiency of single‐molecule junctions, showing that changing the contact groups from 4‐pyridyl to 4‐thioanisole inverts the trend of conductance in a series of molecular wires. Using a combined theoretical and experimental approach, we demonstrate here that the choice of anchor group can have severe repercussions on the electrical properties of molecular junctions with identical backbones. Indeed, the chemical function responsible for mechanically, chemically and electrically coupling the molecule to the metallic electrodes of a single‐entity junction is *not so innocent after all*.

## Results and Discussion

We focussed our efforts on compounds having a dithieno[3,2‐*b* : 2′,3′‐*d*]phosphole oxide, which is known for its high stability, pronounced electron‐accepting behaviour via strong lowest unoccupied molecular orbital (LUMO) stabilisation and exceptional photophysical properties.[^35^, ^36^, ^37^]

**Figure 1:**
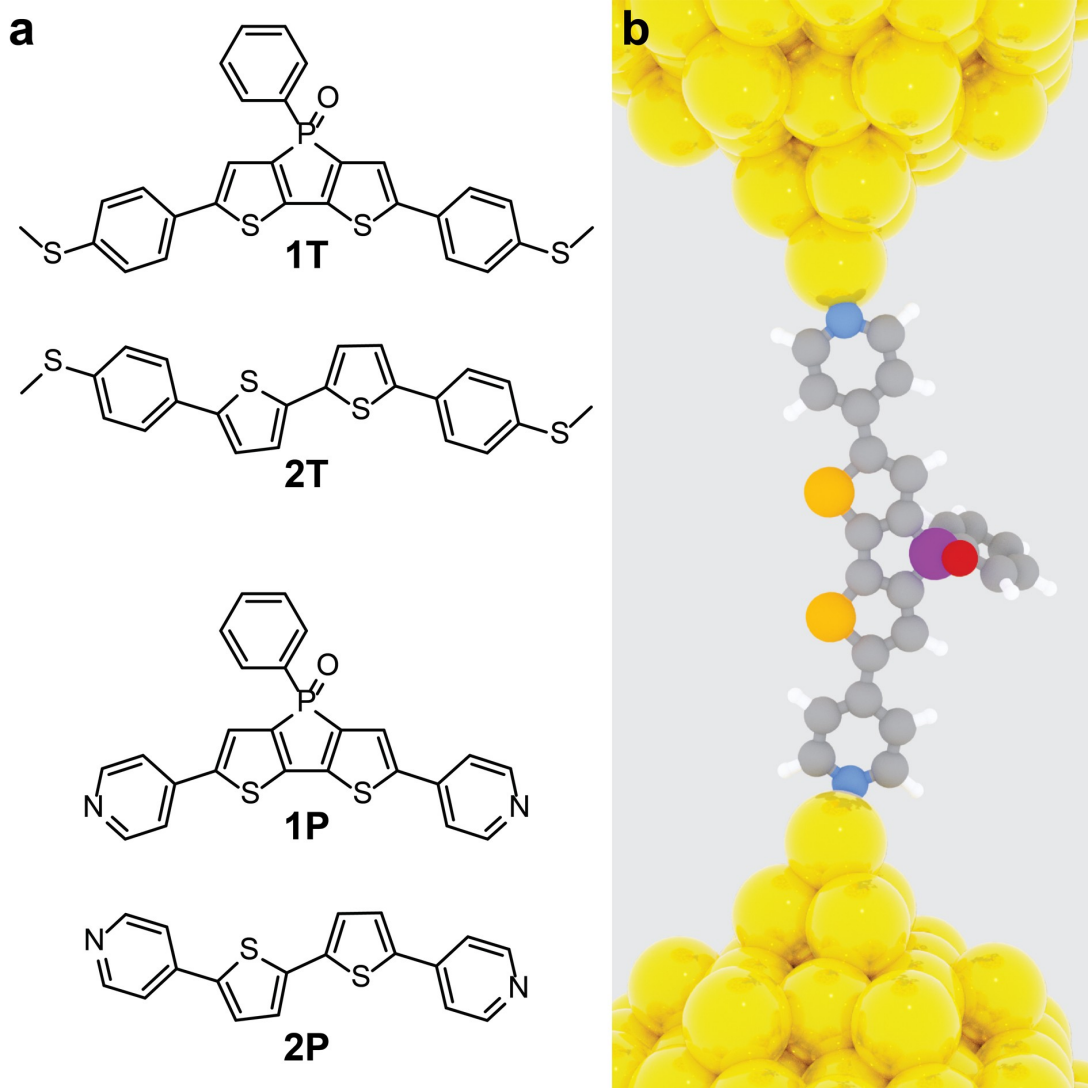
(a) Structures of the molecular wires used in this study and (b) depiction of a single‐molecule junction, with **1P** assembled in the nanogap. Colour legend in (b): C=grey, N=blue, S=orange, P=purple, O=red, Au=yellow, H=white.

Given these outstanding characteristics, it presents an electronic structure uniquely suited to be dramatically affected by the two flanking, conjugated anchor groups. We prepared the dithienophosphole P‐oxides **1T** and **1P** ^[38]^ (Figure 1a) by Suzuki or Stille coupling between 2,6‐dibromo‐dithieno[3,2‐*b* : 2′,3′‐*d*]phosphole oxide and either 4‐(methylthio)phenylboronic acid or (4‐trimethylstannyl)pyridine as per our previously reported general synthetic strategies.[^37^, ^38^] For comparison, we also prepared **2T** and **2P** (Figure 1a), which have similar backbone but they lack the phosphoryl bridge between the two thienylene units. These compounds were prepared by Suzuki coupling of 5,5′‐dibromo‐2,2′‐bithiophene with either 4‐(methylthio)phenylboronic acid or 4‐(pyridyl)boronic acid. The synthetic procedures to **1P**, **2P**, and **2T** are reported elsewhere,[^38^, ^39^] while details about the preparation and characterisation of **1T** can be found in the Supporting Information.

We then used the *scanning tunnelling microscope*—*break junction* (STMBJ) technique^[40]^ to fabricate and characterise molecular junctions (Figure 1b) with these compounds. In this technique, a Au STM tip is moved into and out of contact with a Au substrate at fixed speed (20 nm s^−1^) under electrical bias (300 mV) and in the presence of the target molecule as a 1  mM solution in mesitylene. As the tip crashes into the substrate, a microcontact having conductance $\gg G_{0}$ is generated. When the tip is slowly retracted, this microcontact is stretched and thinned down to an atomic point contact, which is eventually ruptured to yield two atomically sharp Au tips. Molecules present in solution can self‐assemble in the freshly formed gap, thus generating the single‐molecule junction. The tip is further withdrawn to stretch the junction to its most extended state, and then ruptured. The process is repeated thousands of times to obtain data that converges sufficiently in distribution, which is compiled into 1D histograms (yielding a distribution of conductance values) and 2D density plots (showing the correlation between conductance and electrode distance), from which the most probable values are estimated by Gaussian fitting. Details on the equipment and data analysis routines used in this study can be found in our previous publications[^5^, ^41^] and in the Supporting Information.

**Figure 2:**
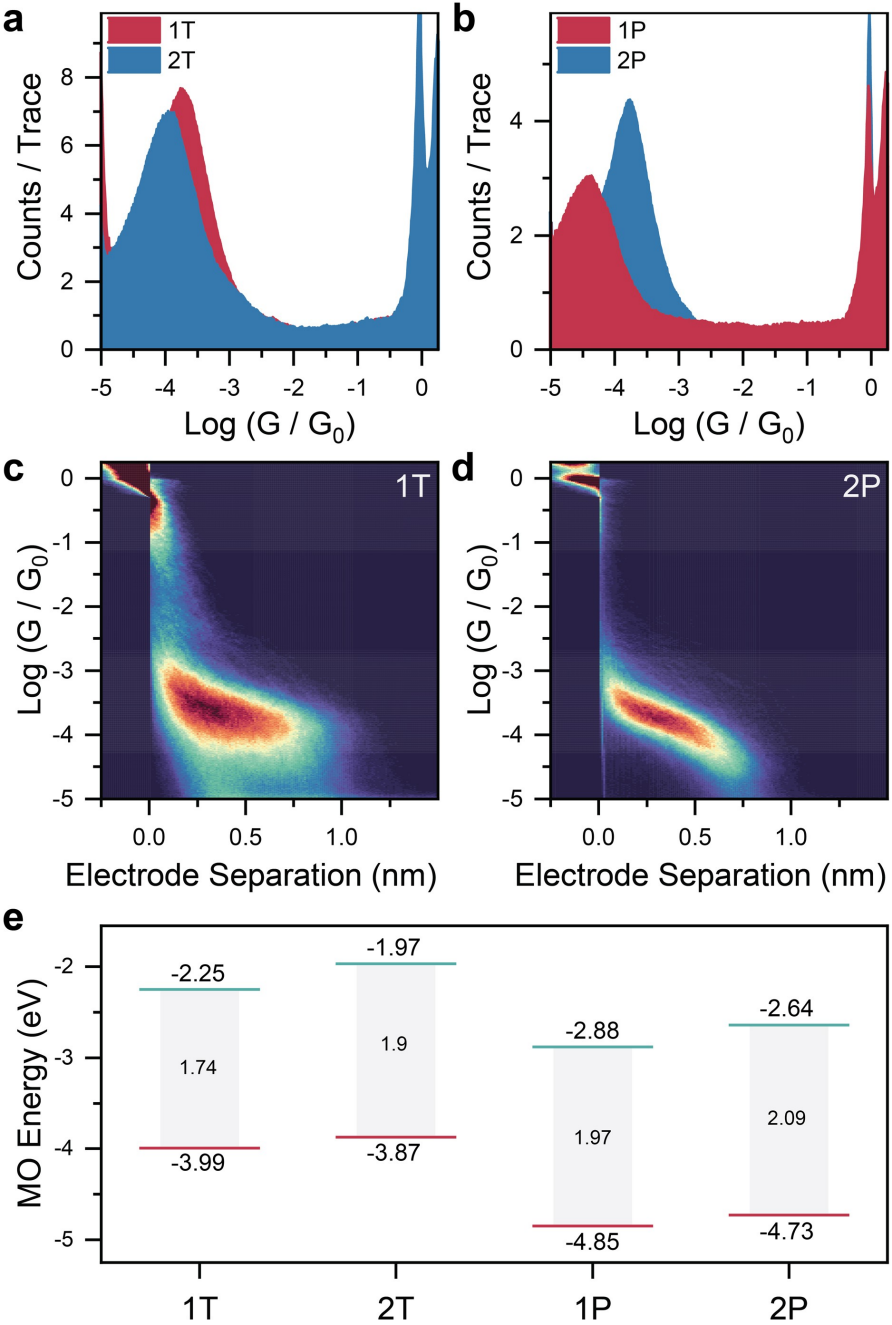
STMBJ experiments and energy diagram. (a) Conductance histograms for the thioanisole‐capped compounds **1T** and **2T**. (b) Conductance histograms for the pyridyl‐capped compounds **1P** and **2P**. (c) 2D density map for **1T**. (d) 2D density map for **2P**. (e) Calculated HOMO and LUMO energy levels for the compounds used in this study. All data was acquired at 300mV source‐drain bias, in a 1 mM solution of the target molecule in mesitylene, with the following statistics: **1T**=8117 traces; **2T**=6316 traces; **1P**=5014 traces, **2P**=7546 traces. Histograms in (a) and (b) are normalised to the number of traces used to compile them. Histograms and 2D plots compiled with 100 bins/decade and 100 bins/nm.

From Figure 2, it can be seen that for the thioanisole‐capped series, **1T** is more conductive than **2T** (Figure 2a), while in the pyridyl‐terminated compounds **1P** and **2P** (Figure 2b) the order is reversed. Analysis of the 2D maps (Figure 2c, and Figure 2d, with further details available in the Supporting Information) shows that in all cases charge transport is probed through the extended molecular wire, as the break‐off distance is consistent with the end‐to‐end length of the molecule (**1T**=1.69 nm; **2T**=1.74 nm; **1P**=1.35 nm; **2P**=1.38 nm by MM2 molecular mechanics), accounting for a 0.5 nm snapback of the Au electrodes upon atomic point contact rupture.^[4]^ The thiophene moieties present in all species are therefore not acting as a supporting electrode contact^[5]^ in these compounds (see Supporting Information for electrode separation histograms).

To better understand these unusual charge‐transport properties we performed theoretical modelling using the SIESTA^[42]^ implementation of density functional theory (DFT). Further details on our methodology are available in the Supporting Information. We first calculated the gas‐phase, ground‐state geometry, and the electronic structure for all compounds, thereby obtaining an energy diagram (Figure 2e). The SIESTA implementation of DFT is known to underestimate the HOMO–LUMO gap (hence the HOMO lying above the Fermi energy of Au in all calculations),^[43]^ and an energy diagram obtained at the B3LYP/6‐31G* level of theory is available in the Supporting Information. Although the frontier orbitals for all compounds are spatially similar (see Supporting Information for isosurface plots), their energies and the resulting HOMO–LUMO gaps are different. In both cases, however, the phosphole‐based compounds (**1T** and **1P**) have lower energies and smaller band gaps than the corresponding bithiophene molecules (**2T** and **2P**), therefore changes in the simple electronic structure of the wires cannot account for the observed changes in the trend of charge‐transport efficiency. We then calculated the electron‐ transport properties of the compounds presented in Figure 1a, sandwiched between two Au electrodes. We computed the mean‐field Hamiltonian of the junctions from the ground‐state relaxed geometry and used the GOLLUM[^44^, ^45^] transport code to calculate the transmission coefficient $T\left(E\right)$ of electrons with energy E passing from one electrode to the other through the molecule. We then used the Landauer formula to calculate the conductance of the junction (see Supporting Information for further details).

**Figure 3:**
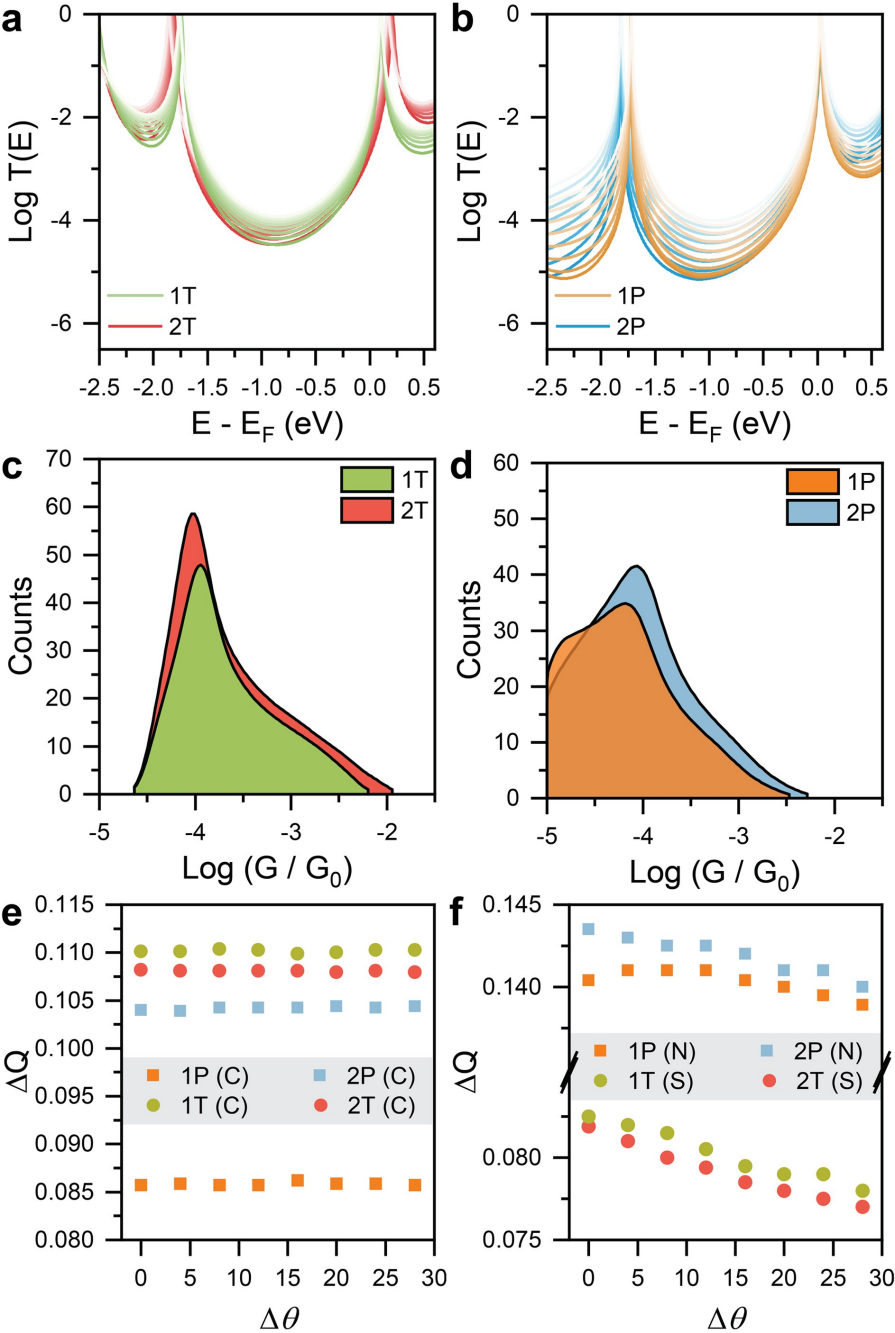
DFT results. Transmission coefficients for **1T**/**2T** (a) and **1P**/**2P** (b) over a range of molecule‐electrode dihedral angles. The T(E) for the ground‐state structure (see Supporting Information for more information) is reproduced in dark colour, and those pertaining to compressed junction (at increases of dihedral angle $\Delta \theta =4^{\circ }$ ) are shown in increasingly lighter colour. Calculated ensemble‐averaged room temperature conductance histograms for **1T**/**2T** (c) and **1P**/**2P** (d). Overall change in charge density on all C atoms of the junction (e) and on the contact atoms N or S (f) as a function of the dihedral angle (where $\Delta \theta =0^{\circ }$ is the ground‐state junction structure) for the four molecules investigated. The charge transfer is from the electrodes to the molecules in all cases.

Our results are presented in Figure 3. To accurately model a STMBJ experiment, where a fresh nanogap of unknown atomic structure is generated at each crash/withdrawal cycle, we considered a range of different junction configurations, obtained by changing the angle between the molecules and the gold electrodes in a range chosen to simulate the geometries probed in a *STMBJ* experiments (more details including structures of the junctions are available in the Supporting Information). We then calculated $T\left(E\right)$ for the full range of structures obtained. The DFT Fermi energy (*E*−*E_F_=*0 eV) is closer to the LUMO resonances for all molecules. This is usually expected when using 4‐pyridyl anchors^[46]^ while for thioanisole/‐SMe contacts, the relative position of the Fermi level can vary with the electron density in the molecular backbone.^[29]^ The transport gap is smaller for **1T** and **1P** than for the respective **2T** and **2P**, in agreement with the gas‐phase energy gaps (more details in the Supporting Information). Compression of the junctions leads to changes in the transport gap, especially visible in the **1T**/**2T** system. (Figure 3a). These phenomena are due to increasing Au−S or Au−N orbital overlap as the molecule/electrode angle is reduced. By reducing the angle, the width of transport resonances increases (Figure 3a and 3b), indicative of greater coupling between the electrode and the molecule which leads to higher overall transmission in the HOMO–LUMO gap.

Using these $T\left(E\right)$ traces, we therefore calculated the ensemble averaged electrical conductance histograms for each compound (Figure 3c and Figure 3d). Accounting for the inaccuracy of DFT at predicting the correct value for the Fermi level of the electrodes,[^47^, ^48^, ^49^] we considered a finite portion of the HOMO–LUMO gap representative of the experimentally‐applied bias[^5^, ^50^] rather than the single DFT‐predicted value of $G\left(E_{F}\right)$ to extract information about the junctions we modelled. The calculated histograms^[51]^ are in qualitative agreement with the experimental findings, predicting $G_{1T}>G_{2T}$ and $G_{1P}<G_{2P}$ . Having established that DFT is able to model the observed behaviour, we then turned our attention to a deeper understanding of the underlying physical mechanism.

The variations of charge density ΔQ (change in charge density upon junction fabrication, e.g., with and without electrode; details in the Supporting Information) on the carbon atoms of the molecular wires are reproduced in Figure 3e, for all the different structures used in the DFT calculations. The strong electron‐accepting properties of dithienophosphole oxide moieties^[25]^ help us in rationalising our findings. In the case of **1T** and **2T**, the electron‐rich thioanisole acts as a “buffer” against the loss of charge density on the molecular backbone, and the overall charge transfer from the electrode is smaller compared to that of **1P** and **2P** (Figure S16) leading to a small difference in ΔQ on the C atoms between **1T** and **2T** (green/red circles in Figure 3e). Intramolecular charge reorganisation is a well‐known phenomenon in donor‐acceptor‐donor molecules, which grants them unique and highly sought‐after optoelectronic properties.[^52^, ^53^, ^54^] The “buffer” effect is absent in the pyridyl‐capped molecular wires, and differences in ΔQ between 1P and **2P** (orange/blue squares in Figure 3e) are significant. For all systems, ΔQ on the carbon atoms does not change across the series of junction geometries investigated, as expected. We then turned our attention to understanding how these subtle changes in the electronic structure of the molecular backbone influence the interface to the electrodes. The variations of charge density on the aurophilic termini of the molecular wire (S for **1T**/**2T** and N for **1P**/**2P**) are reproduced in Figure 3f. While for **1T**, **2T** and **2P** deviations from the ground‐state structure (molecule in the junction) reduces the overall amount of charge transferred, **1P** shows a non‐monotonic trend, and the overall ΔQ is consistently lower than that observed for **2P**. The poor dependency of ΔQ on junction configuration observed in **1P** is indicative of a perturbed interface and a weaker molecule‐electrode coupling. Compounds **1T** and **2T** indeed show similar values of ΔQ and similar charge‐transport efficiency. In the **1P**/**2P** system, however, the already electron‐deficient pyridyl contact is heavily affected by the electron‐accepting behaviour of the dithienophosphole oxide moiety. Charge variations in **1P** are much lower than in **2P** and show little dependence on junction geometry, indicative of a weakened Au−N coordination. All considered, we attribute the observed drop in conductance between **2P** and **1P** to the inability of the pyridyl anchors to mitigate the electron‐withdrawing effect of the dithienophosphole oxide moiety, which result in a less electronically transparent molecule/electrode interface. Small changes in charge density of single atoms, magnified by their propagation to the exquisitely sensitive electrode interface, lead to the significant suppression of charge‐transport efficiency observed in **1P** and the reversal of conductance trends when comparing the T and P series.

## Conclusion

We demonstrated here that the chemical group used to interface a molecule with the two metallic electrodes of a single‐molecule junction must be carefully chosen, as the transparency and energetic alignment of its interface to the metallic electrodes is perturbed by the electronic structure of the conductive backbone. Subtle variations in the charge distribution of the molecular backbone are magnified as they propagate to the exquisitely sensitive molecule/electrode interface, with dramatic effects on molecular conductance. Our results show that internal charge reorganisation provides a strategy to mitigate detrimental effects on charge‐transport efficiency, a phenomenon particularly prominent in the series of dithieno[3,2‐*b* : 2′,3′‐*d*]phosphole oxides we employed in this study, owing to their strong electron‐accepting character. Our results therefore provide a fundamental framework for the design of molecules showing efficient charge‐transport behaviour, especially valid for extremely electron‐rich[^55^, ^56^] or electron‐deficient[^57^, ^58^, ^59^] backbones, which have now found widespread uses as building blocks in supramolecular^[60]^ electronics studies of charge‐transfer[^30^, ^61^] or host–guest[^62^, ^63^, ^64^] complexes.

## Conflict of interest

There are no conflicts to declare.

1

## Supporting information

As a service to our authors and readers, this journal provides supporting information supplied by the authors. Such materials are peer reviewed and may be re‐organized for online delivery, but are not copy‐edited or typeset. Technical support issues arising from supporting information (other than missing files) should be addressed to the authors.

Supporting Information

## Data Availability

Raw STMBJ data for all species discussed in this contribution are available under a CC‐BY license in the University of Liverpool Data Catalogue at DOI: 10.17638/datacat.liverpool.ac.uk/1854.
