# Supplementary material for: Not So Innocent After All: Interfacial Chemistry Determines Charge‐Transport Efficiency in Single‐Molecule Junctions
Source: Angew Chem Int Ed Engl. 2023 May 4;62(24):e202302150. doi: 10.1002/anie.202302150 (PMC10953449; doi:10.1002/anie.202302150)
Supplement: Supplementary file 1 — Supporting Information [file ANIE-62-0-s001.pdf]

Angewandte Chemie
Eine Zeitschrift der Gesellschaft Deutscher Chemiker

Supporting Information

Not So Innocent After All: Interfacial Chemistry Determines Charge-Transport Efficiency in Single-Molecule Junctions

A. Daaoub, J. M. F. Morris, P. Demay-Drouhard, A. Hussein, S. J. Higgins, H. Sadeghi, R. J. Nichols, A. Vezzoli, T. Baumgartner*, S. Sangtarash**

1. Synthetic Details

All manipulations were performed under an argon atmosphere using standard Schlenk techniques. Solvents were dried with an MBraun Solvent Purification System. An oil bath was used for reactions requiring heating. Starting materials and reagents were purchased from Sigma-Aldrich, Oakwood Chemical, or Strem Chemicals and used without further purification. Compounds **1P**,[1] **2P**,[1] and **2T**[2] were synthesized according to previously reported procedures. In addition, compound **2T** was further purified by sublimation. ^{1}H, $^{13}C\{^{1}H\}$, and $^{31}P\{^{1}H\}$ NMR spectra were recorded on a Bruker Avance 400 MHz spectrometer. Chemical shifts were referenced to residual non-deuterated solvent peaks (CD_2Cl_2: 5.32 ppm for ^{1}H and 53.5 ppm for ^{13}C). Mass spectrometry data was provided from the AIMS Mass Spectrometry Laboratory at the University of Toronto. Samples were run in DART positive ion mode, utilizing an AccuTOF 4G instrument. IR spectroscopy was performed on a Bruker Alfa FT-IR Spectrometer using a KBr pellet. Optical spectroscopy was performed on an Agilent Cary 5000 UV-vis spectrophotometer. Fluorescence spectroscopy was performed on an Edinburgh Instruments FS5 spectrofluorometer; quantum yield was measured by direct methods using an integrating sphere. The solid-state structure of **1T** was determined using Bruker DQ Quest eco. The structure was solved and refined using OLEX2 software.

Preparation of 2,6-bis-(4-methylthiophenyl)dithieno-P-phenylphosphole oxide (1T): 2,6-dibromodithieno-P-phenylphosphole oxide (0.50g, 1.1 mmol), tetrakis(triphenylphosphine)palladium (0.13 g, 0.1 mmol) and 4-(methylthio)phenylboronic acid (0.38 g, 2.3 mmol) were combined in toluene (100mL) and aqueous potassium carbonate (2 M, 15mL) and refluxed overnight to give an orange suspension. The mixture was washed with brine, then water and extracted with dichloromethane (4 x 100 mL). The solution was dried with magnesium sulfate, filtered and concentrated under reduced pressure to give an orange solid. The solid was purified by vapour diffusion of diethyl ether (150 mL) into a dichloromethane solution (75 mL) of **1T** at 5 °C over the course of 4 days. The supernatant was decanted from the resulting orange crystals, and the recrystallization process was repeated once. Single crystals suitable for X-ray diffraction studies were grown by vapor diffusion of an acetone solution of **1T** into acetonitrile at -15 °C over the course of three days. Yield = 0.27 g, 45 %. ^{1}H NMR (400.34 MHz, CD_2Cl_2): δ = 7.78 (2H, dd, $^{3}J_{HH}$ = 12 Hz, $^{3}J_{HH}$ = 8 Hz), 7.52 (7H, m), 7.34 (2H, br), 7.26 (4H, d, $^{3}J_{HH}$ = 8 Hz), 2.50 (6H, s) ppm; ^{13}C NMR (100.67 MHz, CD_2Cl_2): δ = 148.5 (d, $^{1}J_{CH}$ = 20 Hz), 144.3 (d, $^{1}J_{CH}$ = 20 Hz), 139.8 (d, $^{1}J_{CP}$ = 111 Hz), 139.8 (s), 132.9 (s), 131.2 (d, $^{1}J_{CH}$ = 10 Hz), 130.4 (s), 130.0 (s), 129.4 (d, $^{1}J_{CH}$ = 10 Hz), 126.6 (d, $^{1}J_{CP}$ = 60 Hz), 121.3 (d, $^{1}J_{CH}$ = 20 Hz), 15.7 (s) ppm; $^{31}P\{^{1}H\}$ NMR (162.06 MHz, $CDCl_3$): δ = 19.2 ppm; FT-IR (ranked intensity): ν = 480.0 (6), 521.1 (1), 550.8 (4), 627.3 (5), 682.5 (12), 713.7 (9), 715.1 (8), 737.8 (11), 812.8 (2), 849.6 (15), 1091.8 (10), 1195.2 (7), 1203.6 (3), 1434.5 (13), 1472.7 (14) cm^{-1}; UV-vis absorption: λ_{max} = 444 nm; fluorescence emission: λ_{max} = 556 nm; Φ = 66 %; HRMS: m/z 533.02856 (calculated); 533.02877 (found) $[C_{28}H_{22}OPS_4]^+$ ($[M]^+$).

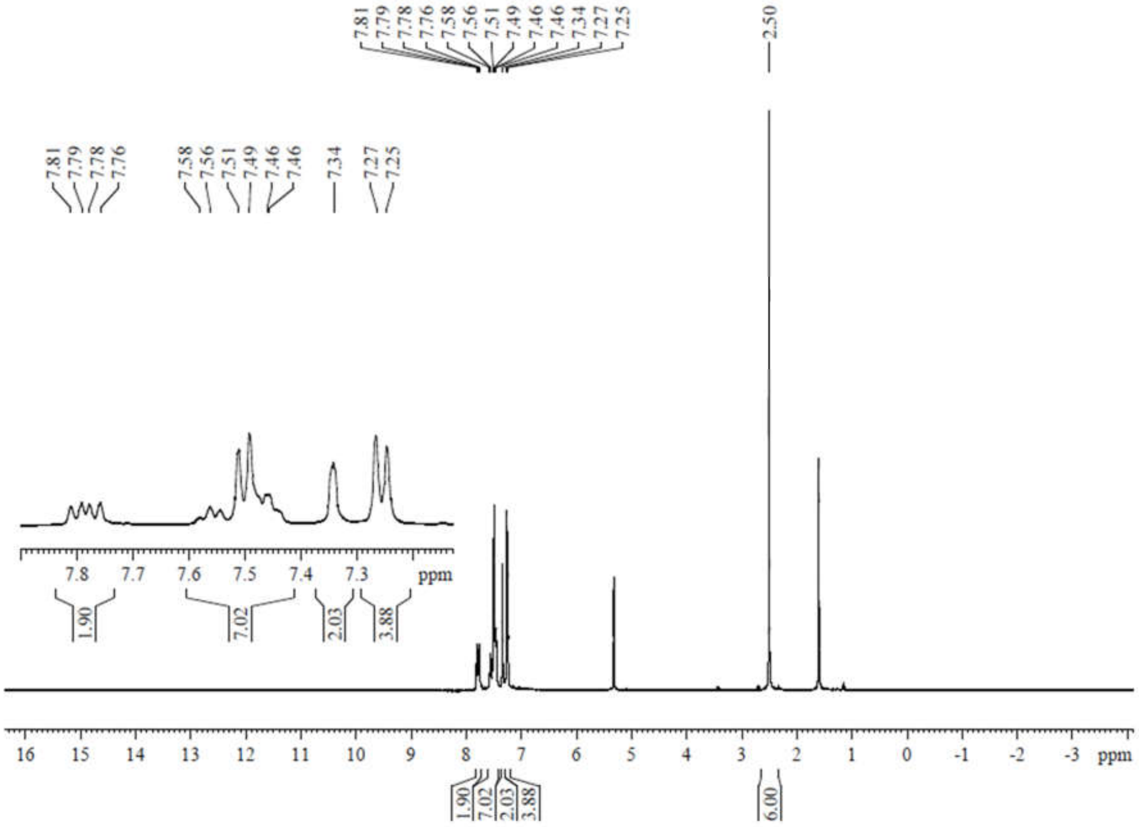

Figure S1. 1H NMR spectrum of 1T in CD2Cl2.

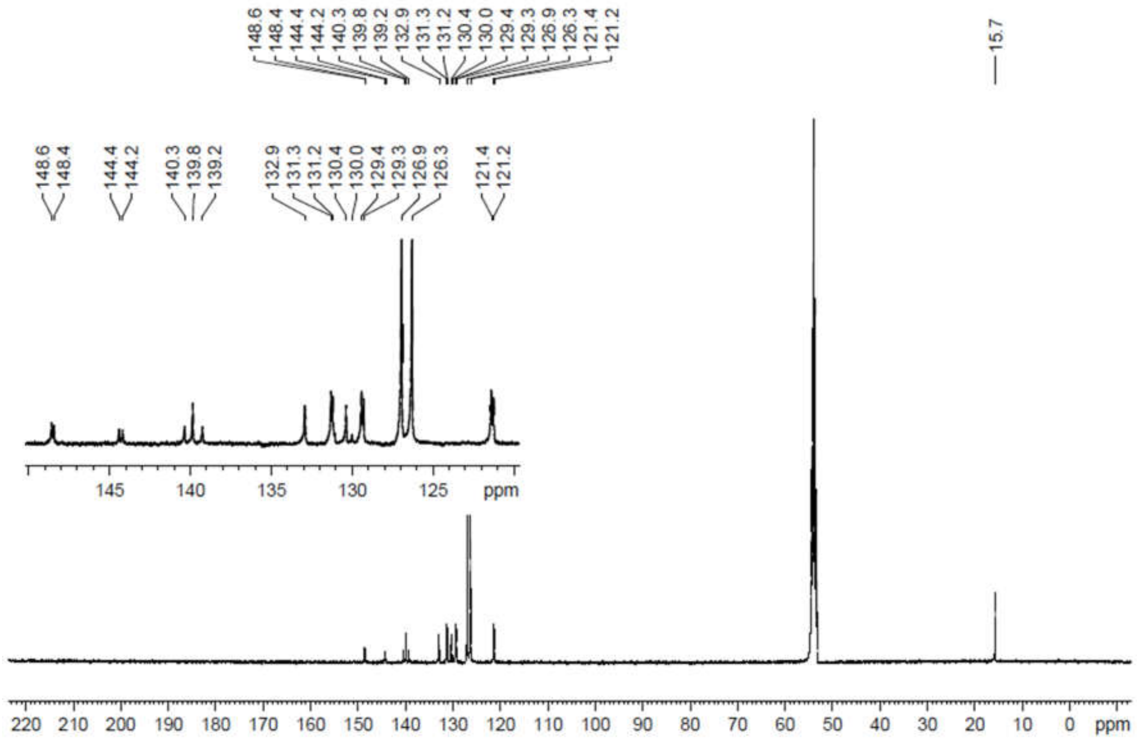

Figure S2. 13C{1H} NMR spectrum of 1T in CD2Cl2

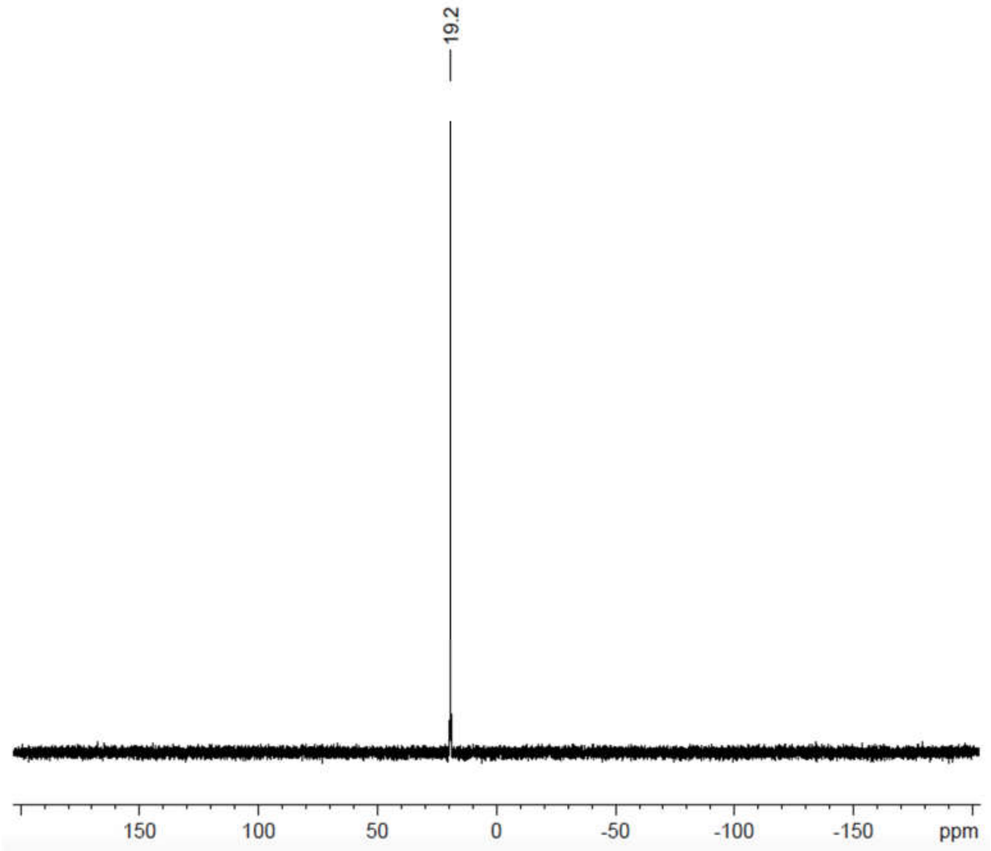

Figure S3. $^{31}P\{^{1}H\}$ NMR spectrum of **1T** in $CDCl_3$.

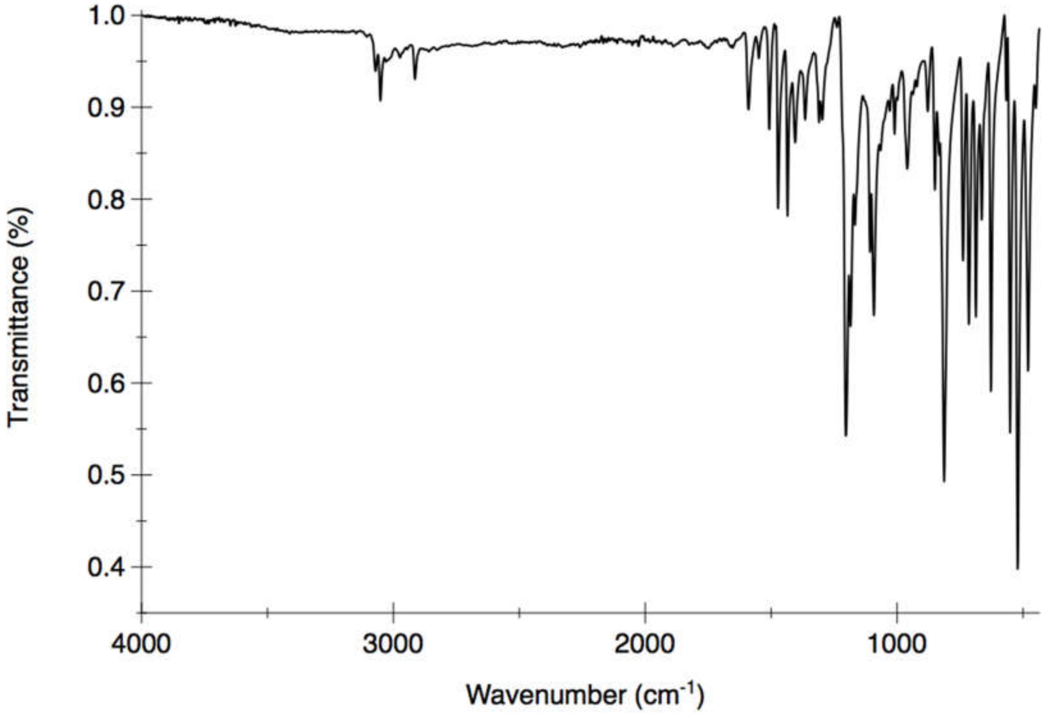

Figure S4. ATR-IR spectrum of **1T**.

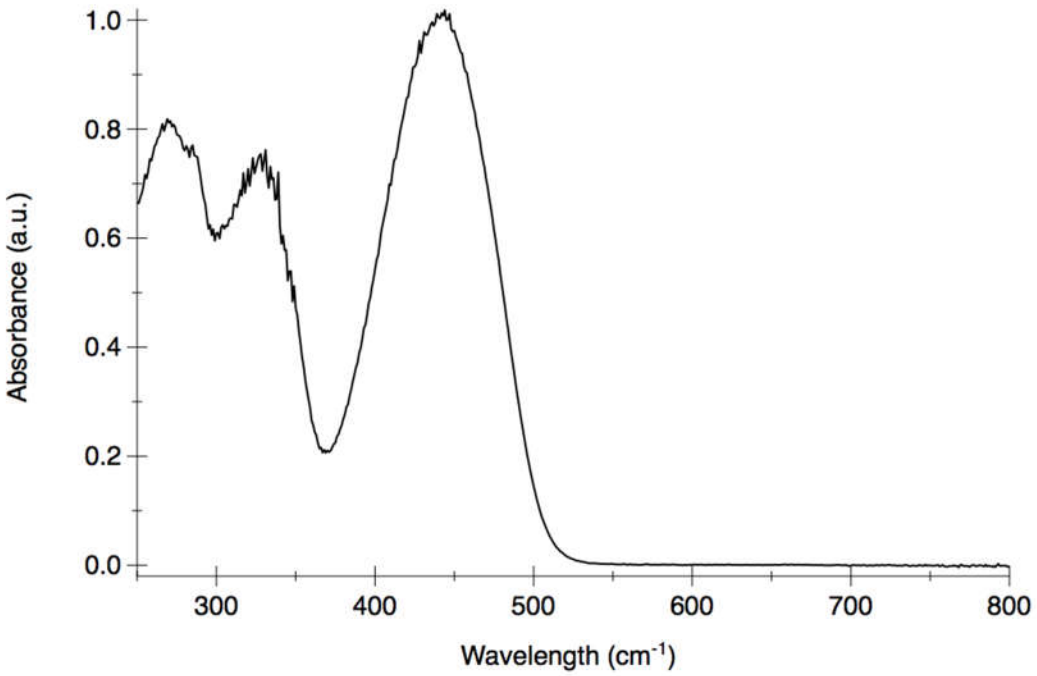

Figure S5. UV-vis absorption spectrum of **1T** in dichloromethane

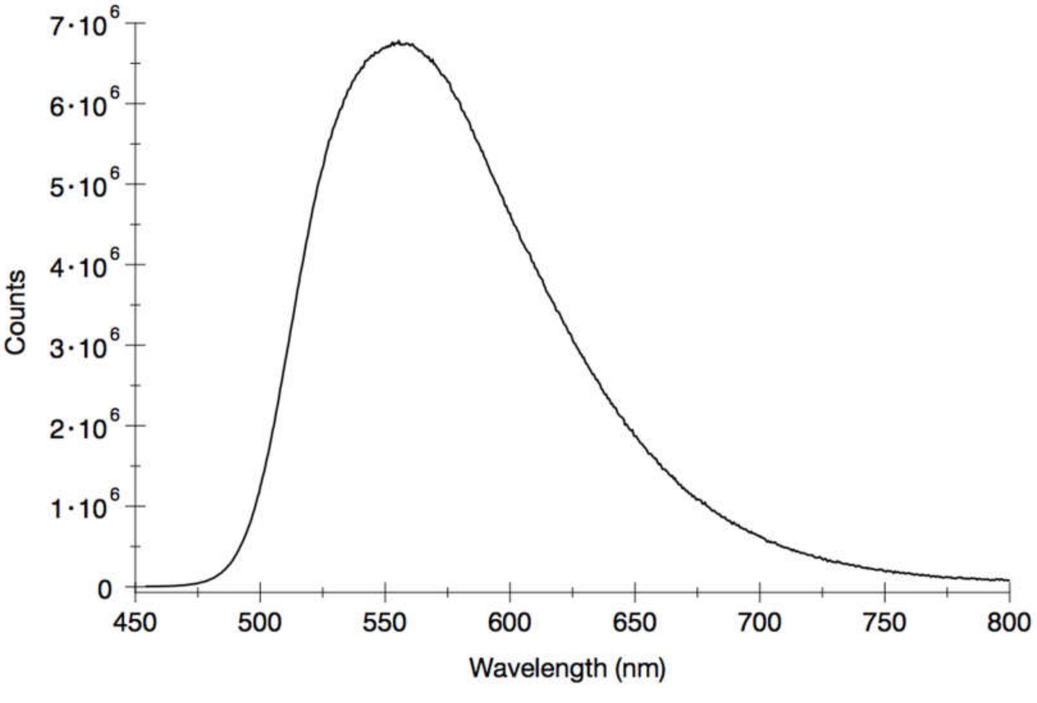

Figure S6. Fluorescence emission spectrum of **1T** in dichloromethane.

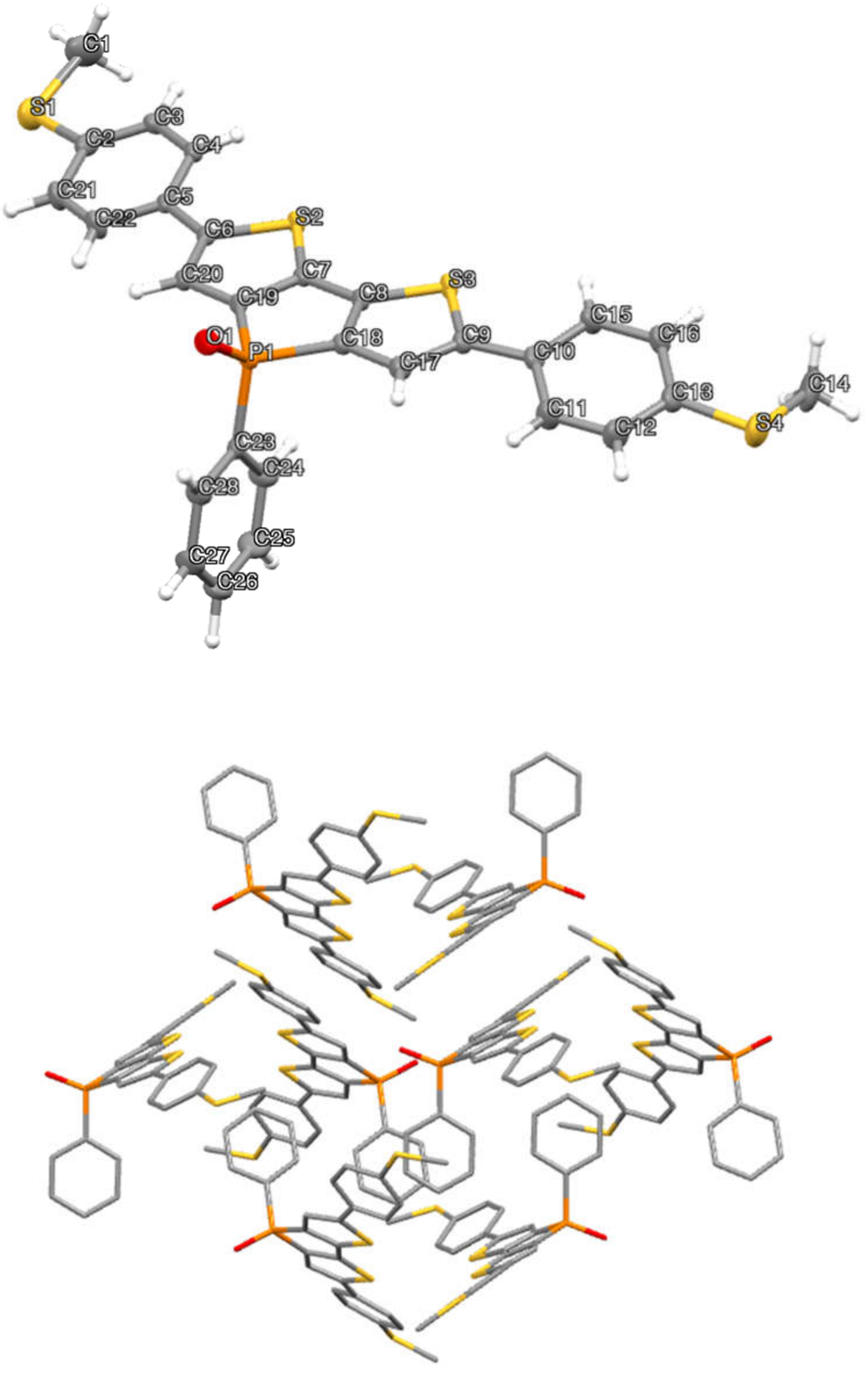

Figure S7. Structure of **1T** in the solid state (50% probability level). Selected bond lengths (Å) and angles (º): P1-C18, 1.812(3); P1-C19, 1.799(3); P1-O1, 1.479(2); S1-C2 1.757(3); S4-C13, 1.759(3); C22-C5-C6-S2, 167.2(2); C11-C10-C9-S3, 163.4(3).

Identification code	VB003_m_2_a
Empirical formula	$C_{28}H_{21}OPS_4$
Formula weight	532.66
Temperature/K	172(2)
Crystal system	monoclinic
Space group	P2/c
a/Å	16.1538(11)
b/Å	12.3259(9)
c/Å	12.7674(9)
α/°	90
β/°	102.276(3)
γ/°	90
Volume/Å^3	2484.0(3)
Z	4
ρ_{calc}g/cm^3	1.424
μ/mm^1	0.468
F(000)	1104.0
Crystal size/mm^3	0.1 × 0.1 × 0.1
Radiation	Mo Kα (λ = 0.71073)
2Θ range for data collection/°	4.646 to 66.792
Index ranges	$-24 \leq h \leq 24$, $-19 \leq k \leq 18$, $-19 \leq l \leq 19$
Reflections collected	98270
Independent reflections	9558 [R_{int} = 0.1231, R_{sigma} = 0.0795]
Data/restraints/parameters	9558/0/391
Goodness-of-fit on F^2	1.181
Final R indexes [I>=2σ (I)]	R_1 = 0.0962, wR_2 = 0.1550
Final R indexes [all data]	R_1 = 0.1426, wR_2 = 0.1701
Largest diff. peak/hole / e Å^{-3}	0.68/-0.57
CCDC #	2215946

2. Single-Molecule Conductance Measurements

2.1 Instrument details

We employed a modified Molecular Imaging PicoScan2500 for the fabrication and characterisation of single-molecule junctions. Its design is based on that of a similar instrument (a modified Keysight 5500 SPM system) we discussed in our previous publications.[3,4] We built a bespoke break-out box to access the STM signals and apply a custom source-drain bias and a custom voltage ramp to the Z piezoelectric transducer using two DB25 pinout boards (Phoenix Contact VS25BU-DSUB/25-MPT-05). Signals are imposed with a Keysight 33522B arbitrary waveform generator (AWG). The system is also equipped with a Thorlabs HVA200 high-voltage amplifier (not used in this study) which extend the Z piezo range beyond the ±10 V (corresponding to ±13.6 nm) offered by the Keysight 33522B AWG. Current is monitored with a Femto DLPCA-200 single-channel transimpedance amplifier ($10^6\ V/A$ used in this study), and all signals (Z piezo voltage, source-drain bias, Femto OUT) are simultaneously acquired with a 24-bit USB data acquisition system (National Instruments cDAQ-9171 with NI-9239 module) at $20\ kSa/s$. The current signal is fed back into the STM controller through the break-out box to use the in-built proportional-derivative-integral feedback loop for tip approach and imaging of the substrate. A set of resistors between the AWG and the substrate prevents overload of the current preamplifier and limits the current through the system when $G \gg G_0$. In this study, we used a 100 $k\Omega$ resistor for all compounds. The presence of this resistor and the effective bias drop as the conductance of the junction is $> G_0$ allows us to probe conductance over more than 7 orders of magnitude (from $\cong 10^2$ to $\cong 10^{-5.5}\ G_0$ using a single-channel preamplifier, without changing amplification.

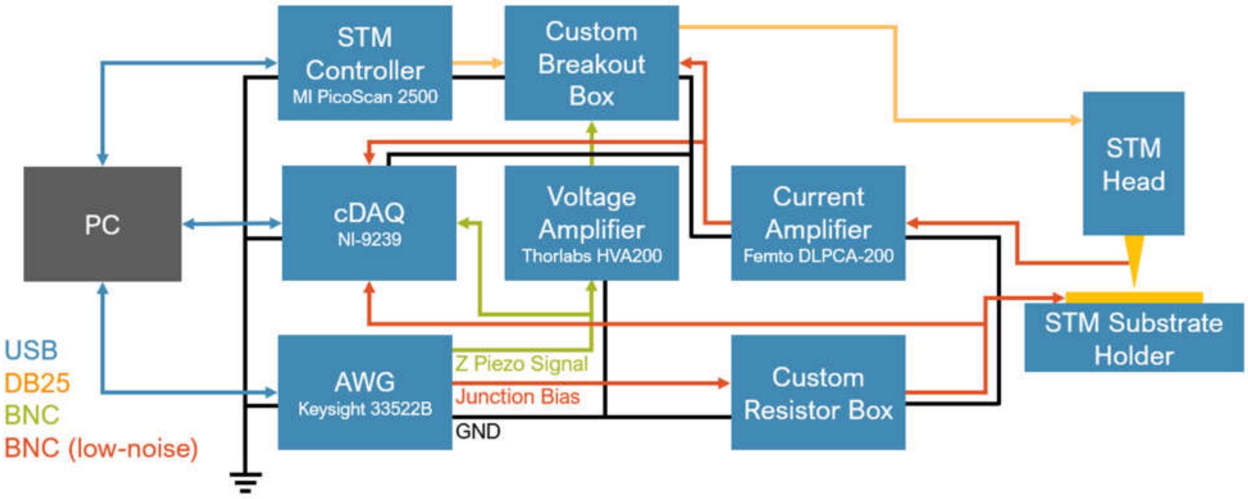

Figure S8. Schematics of the modified MI PicoScan2500 STM used in this study.

All experiments have been performed with a Au tip cut from a spool of Au wire (99.998%, ThermoFisher Scientific PREMION) and substrates prepared by e-beam evaporation (Korvustech Hex PVD Tau) of 100 nm of Au (99.99+% nuggets, Advent Research Materials) on freshly cleaved muscovite mica (Agar Scientific). Substrates were briefly flame-annealed with a butane torch immediately before use. Mesitylene 98+% used in these experiments was purchased from TCI UK and used without further purification.

Data logging was performed through a bespoke Labview VI. In all experiments, the data stream from the three channels (Z piezo voltage, source-drain bias, Femto OUT) is continuously monitored as the tip is repeatedly crashed into the substrate and then withdrawn. The VI converts the piezo signal (in V) into nm using the piezo displacement ratio obtained by calibrating it against the step height of a reconstructed Au(111) surface (currently 13.5 nm/V). The VI also calculates the current from the FemtoOUT ($I = Femto_{OUT} \times 10^{-6}$), and then the conductance ($G = I/V$), as a function of G_0. A trigger/gate function cuts the trace as the tip is crashed, when $G > 10\ G_0$, gating a total of 19000 samples (*i.e.* 19 nm tip withdrawal). The extra nm of tip withdrawal is discarded, and acts as a buffer for the trigger/gate function. This way, the VI outputs an ASCII table for each crash/withdraw cycle, as tip displacement (nm), source-drain bias (V), and junction conductance (G/G_0). An automated algorithm in the acquisition VI filters out traces that did not decay to the noise level of the instrument ($\cong 10^{-5.5}\ G_0$ at 300 mV bias) within the 19 nm Z piezo ramp. If the system repeatedly fails to either reach the noise level when the tip is fully withdrawn or to engage the substrate at $G > 5\ G_0$ at the beginning of the piezo ramp, data acquisition is stopped. The tip is then moved to a different area on the substrate using the XY piezoelectric transducer of the STM, and the measurement is then resumed. All traces collected this way are then used to compile the histograms and 2D density maps shown in the manuscript and here in the SI with no further selection.

2.2 Additional STMBJ Data

We report here 1D histogram and 2D plots for all compounds used in this study.

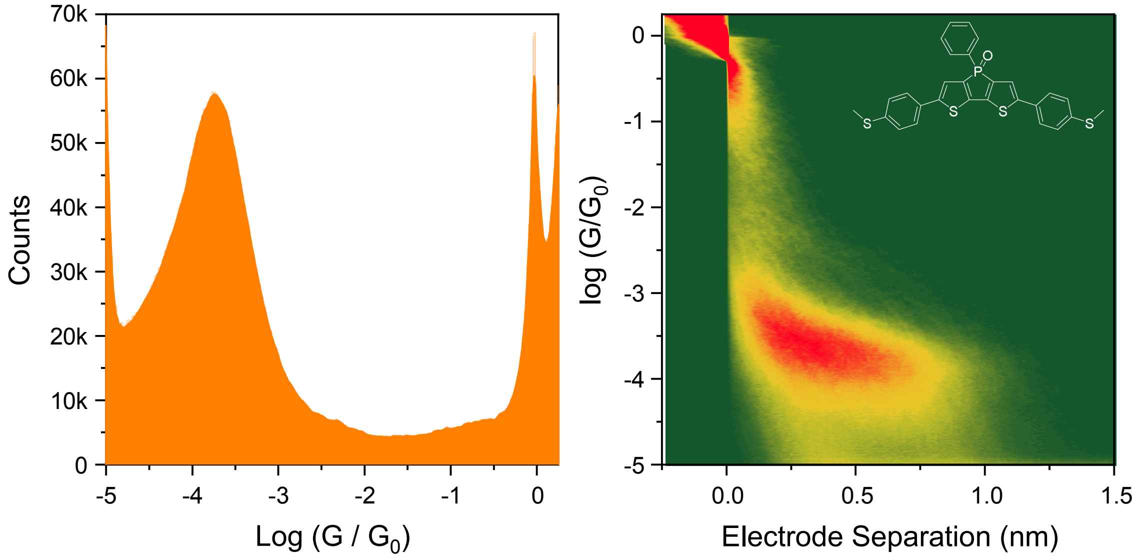

Figure S9. Histogram and 2D plot for **1T**. 300 mV bias, 1mM in mesitylene, 8117 traces, 100 bins/decade, 100 bins/nm.

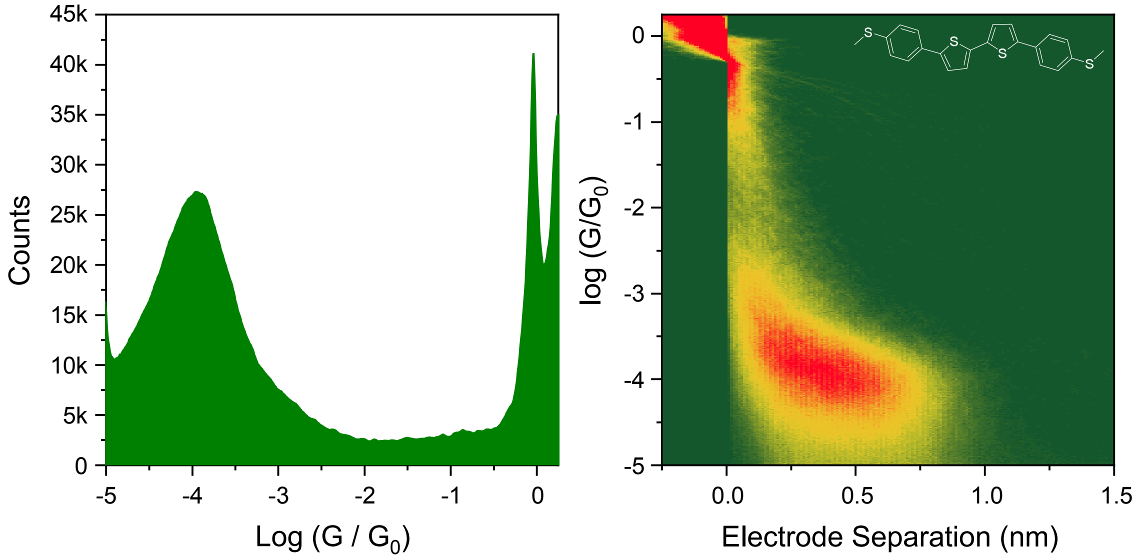

Figure S10. Histogram and 2D plot for **1T**. 300 mV bias, 1mM in mesitylene, 6316 traces, 100 bins/decade, 100 bins/nm.

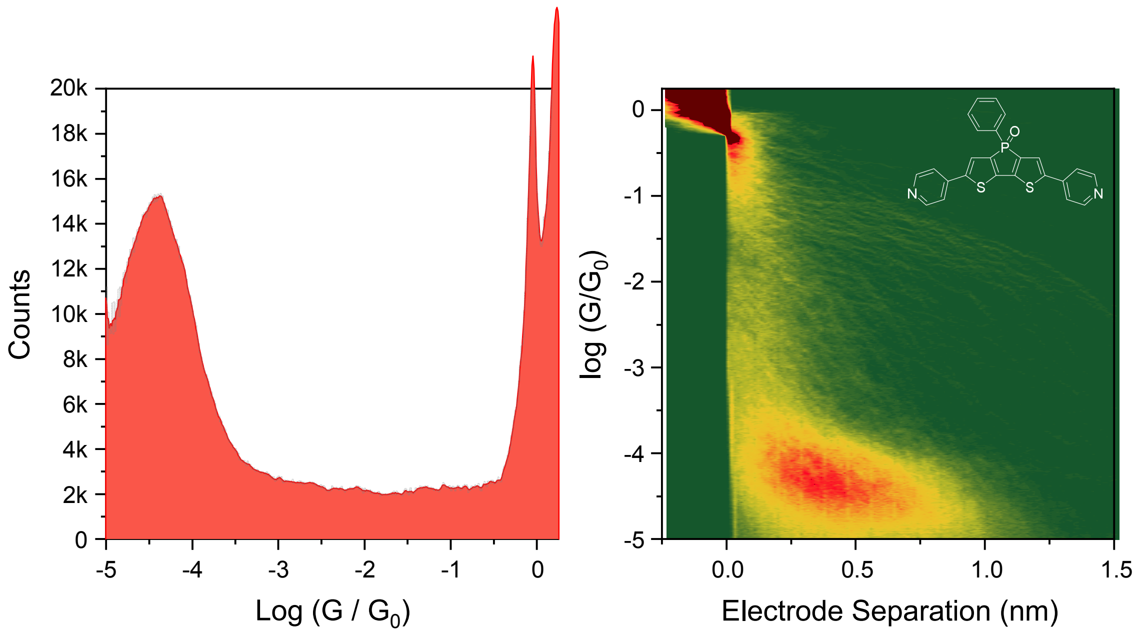

Figure S11. Histogram and 2D plot for **1P**. 300 mV bias, 1mM in mesitylene, 5014 traces, 100 bins/decade, 100 bins/nm.

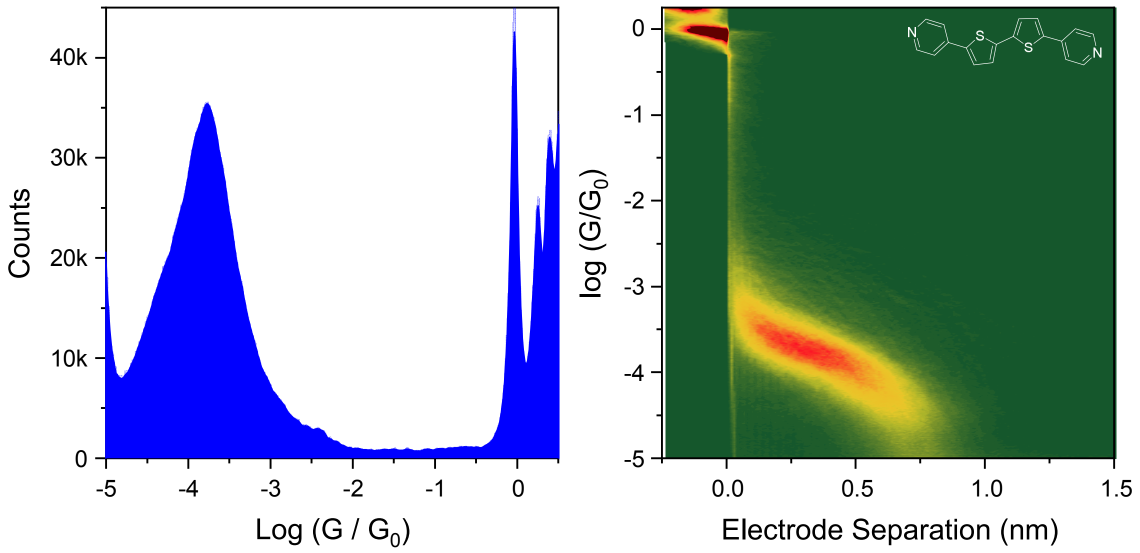

Figure S12. Histogram and 2D plot for **2P**. 300 mV bias, 1mM in mesitylene, 7546 traces, 100 bins/decade, 100 bins/nm.

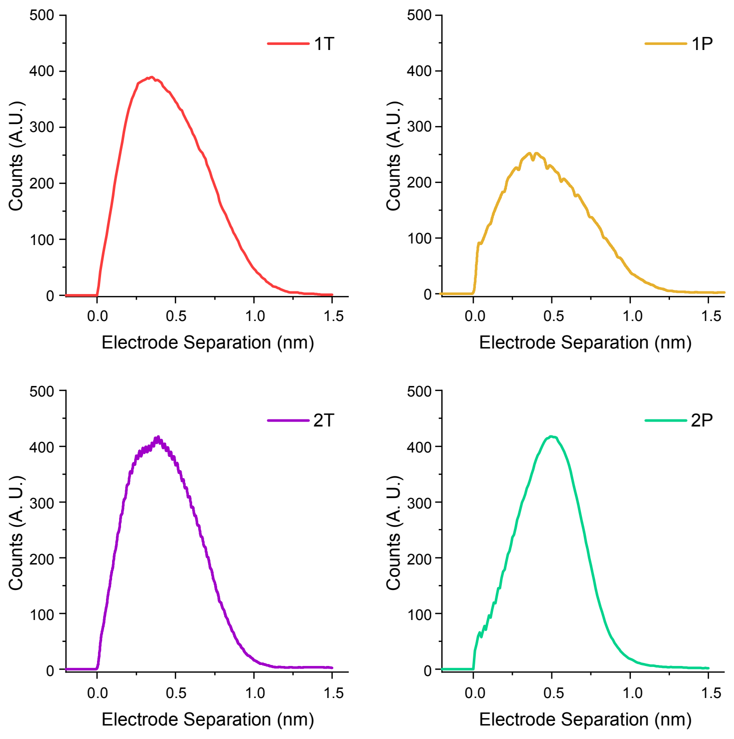

Figure S13. Electrode separation histograms for the four compounds used in this study. The histograms have been compiled from the 2D maps shown in Figure S9-S12, using the datapoints in the G ± σ window determined by Gaussian fitting of the peak in the 1D conductance histogram. Gaussian interpolation of these plots allows us to estimate the 95th percentile of junction extension ($x_c + 2\sigma$): **1T** = 0.97 nm; **2T** = 1.01 nm; **1P** = 1.05 nm; **2P** = 0.98 nm.

3. Additional DFT-NEGF Details

3.1. DFT Methods

The optimized geometry and ground state Hamiltonian and overlap matrix elements of each structure was self-consistently obtained using the SIESTA[5,6] implementation of density functional theory (DFT). SIESTA employs norm-conserving pseudo-potentials to account for the core electrons and linear combinations of atomic orbitals to construct the valence states. The local density approximation (GGA) of the exchange and correlation functional is used with CA parameterization, a double-ζ polarized (DZP) basis set, a real-space grid defined with an equivalent energy cut-off of 250 Ry. The geometry optimization for each structure is performed to the forces smaller than 10 meV/Å.

The mean-field Hamiltonian obtained from the converged DFT calculation was combined with the GOLLUM[7] implementation of the non-equilibrium Green's function method to calculate the phase-coherent, elastic scattering properties of the each system consist of left gold (source) and right gold (drain) leads and the scattering region. The transmission coefficient *T(E)* for electrons of energy E (passing from the source to the drain) is calculated via the relation: $T(E) = Trace\big(\Gamma_R(E)G^R(E)\Gamma_L(E)G^{R\dagger}(E)\big)$. In this expression, $\Gamma_{L,R}(E) = i\left(\sum_{L,R}(E) - \sum_{L,R}{}^{\dagger}(E)\right)$ describe the level broadening due to the coupling between left (L) and right (R) electrodes and the central scattering region, $\sum_{L,R}(E)$ are the retarded self-energies associated with this coupling and $G^R = (ES - H - \sum_L - \sum_R)^{-1}$ is the retarded Green's function.

The electrical conductance is then calculated using the Landauer formula $G(E_F, T) = G_0 L_0$, $L_n = \int_{-\infty}^{+\infty} dE\,(E - E_F)^n\, T(E)(-\partial f(E, T, E_F)/\partial E)$ and $f = (e^{(E-E_F)/k_B T} + 1)^{-1}$ is the Fermi-Dirac probability distribution function, T is the temperature, E_F is the Fermi energy, $G_0 = 2e^2/h$ is the conductance quantum, e is electron charge and h is the Planck's constant.

To calculate the distribution of charges on each molecule and their redistribution according to molecular conformations, we calculate the Mulliken charge on each atom for each conformation using DFT. The difference in charge is calculated as $\Delta Q\ = |Q_n - Q_0|$ where Q_n is the Mulliken charge on a given atom or group of atoms for the configuration n (between the gold electrodes) and Q_0 is the Mulliken charge at the same atom(s) in the ground state configuration of the isolated molecule (without the electrodes).

Each gold electrode includes 124 gold atoms: 4 on the electrode tip and 120 on the bulk of each gold electrode. We leave 40 atoms as buffer layer between the tip and the bulk gold electrode to ensure that the electrode electronic structure is not affected by different molecules. The main difference in charge happens at the interface gold atoms (on the tip of electrode) shown in Fig. S16. As a result of this, the position of the frontier orbitals energy with respect to the Fermi energy of electrodes is modified.

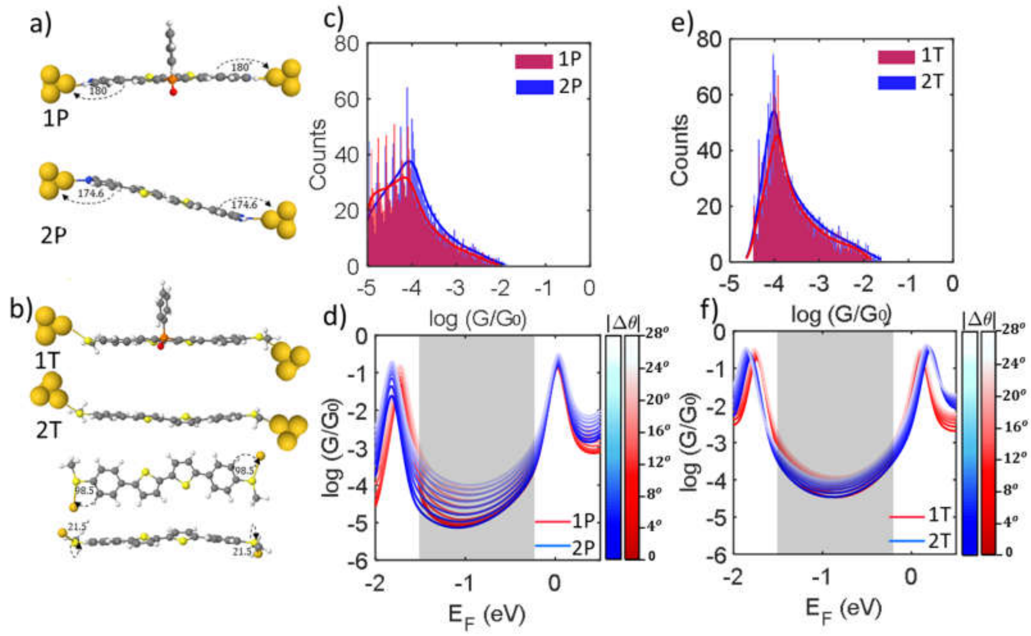

Figure S14. (a, b) Ground-state structure of the molecules **(1P,2P)** and **(1T,2T)** between Au electrodes. (c,e) Calculated electrical conductance histograms of the molecules **(1P,1T)** (red) and **(2P,2T)** (blue) at grey region. (d,f) Electrical conductance for corresponding molecules for a range of configurations with different dihedral angles ($|\Delta\theta| = 4^o$).

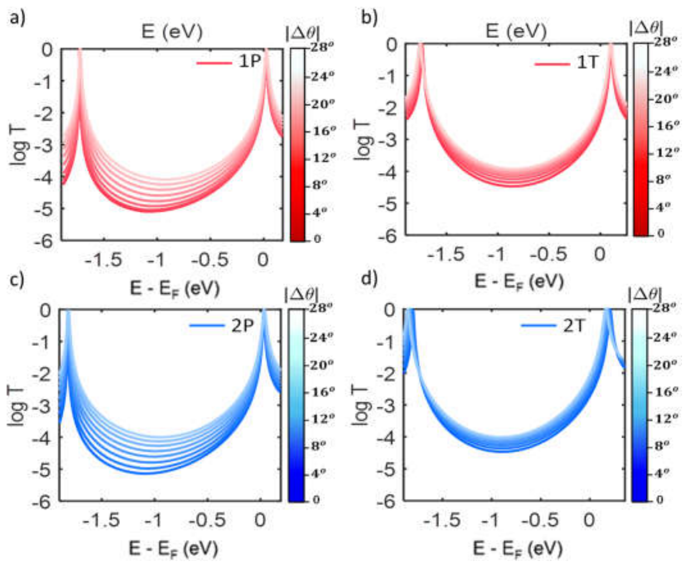

Figure S15. (a- d) Transmission coefficients of the molecules (**1P**,**1T**) (red) and (**2P**,**2T**) (blue) for a range of configurations with different dihedral angles ($|\Delta\theta| = 4^o$).

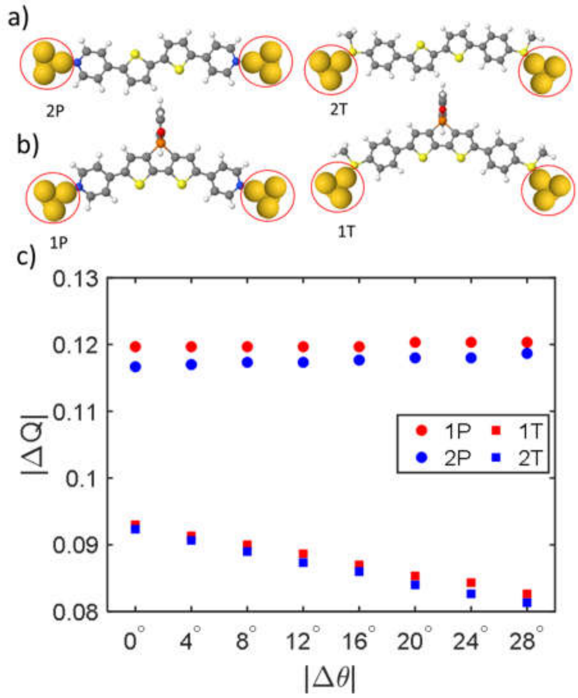

Figure S16. Charge density on the tip electrode atoms. (a,b) Structure of the molecules (**1P**, **2P)** and (**1T**, **2T**) between Au electrodes for a range of configurations with different dihedral angles ($|\Delta\theta| = 4^o$). (c) Electron charge density on tip electrode (3Au red circle) that connected with (**1P**, **2P)** and (**1T**, **2T**) as a function of the dihedral angle between the molecule and the Au electrodes. The charge transfer is from the electrodes to the molecules in all cases.

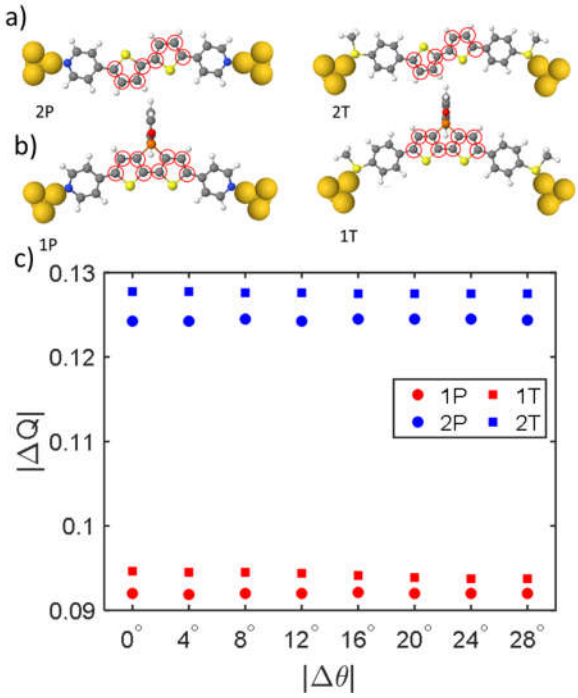

Figure S17. Charge density on carbon atoms. (a,b) Structure of the molecules (**1P**, **2P)** and (**1T**, **2T**) between Au electrodes for a range of configurations with different dihedral angles ($|\Delta\theta| = 4^o$). (c) Electron charge density on carbon atoms (red circle) in (**1P**,**2P)** and (**1T**, **2T**) as a function of the dihedral angle between the molecule and the Au electrodes.

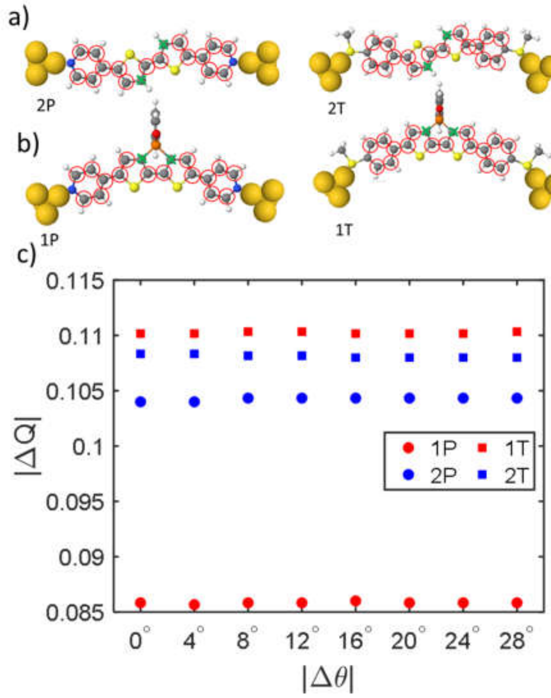

Figure S18. Charge density on carbon atoms. (a,b) Structure of the molecules (**1P**, **2P)** and (**1T**, **2T**) between Au electrodes for a range of configurations with different dihedral angles ($|\Delta\theta| = 4^o$). (c) Electron charge density on carbon atoms (red circle) in (**1P**, **2P)** and (**1T**, **2T**) as a function of the dihedral angle between the molecule and the Au electrodes.

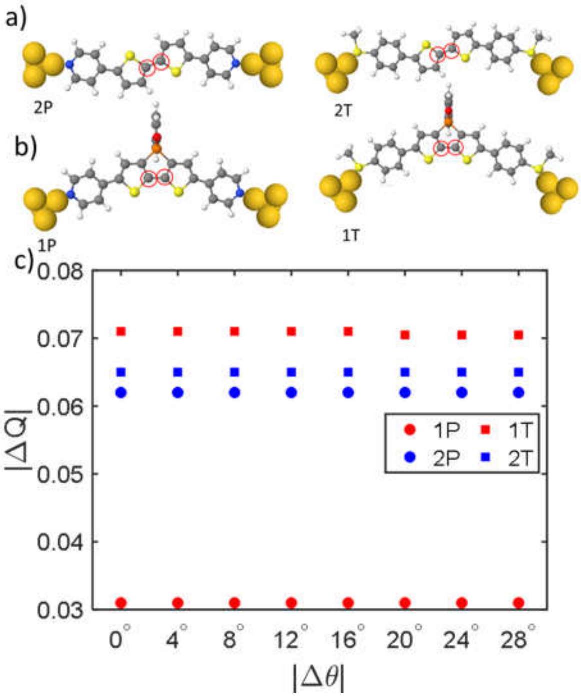

Figure S19. Charge density on carbon atoms. (a,b) Structure of the molecules (**1P**, **2P)** and (**1T**, **2T**) between Au electrodes for a range of configurations with different dihedral angles ($|\Delta\theta| = 4^o$). (c) Electron charge density on carbon atoms (red circle) in (**1P**,**2P)** and (**1T**, **2T**) as a function of the dihedral angle between the molecule and the Au electrodes.

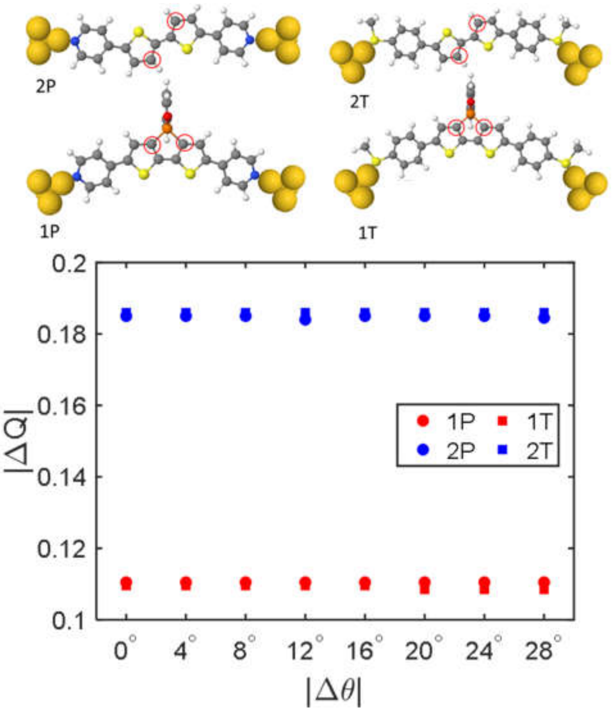

Figure S20. Charge density on carbon atoms. (a,b) Structure of the molecules (**1P**, **2P)** and (**1T**, **2T**) between Au electrodes for a range of configurations with different dihedral angles ($|\Delta\theta| = 4^o$). (c) Electron charge density on carbon atoms (red circle) in (**1P**, **2P)** and (**1T**, **2T**) as a function of the dihedral angle between the molecule and the Au electrodes.

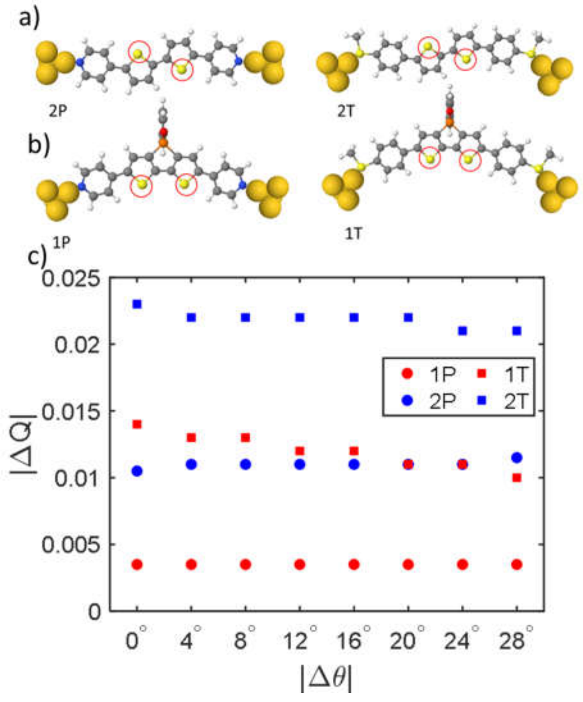

Figure S20. Charge density on sulfur atoms. (a,b) Structure of the molecules (**1P**, **2P)** and (**1T**, **2T**) between Au electrodes for a range of configurations with different dihedral angles ($|\Delta\theta| = 4^o$). (c) Electron charge density on sulfur atoms (red circle) in (**1P**, **2P)** and (**1T**, **2T**) as a function of the dihedral angle between the molecule and the Au electrodes.

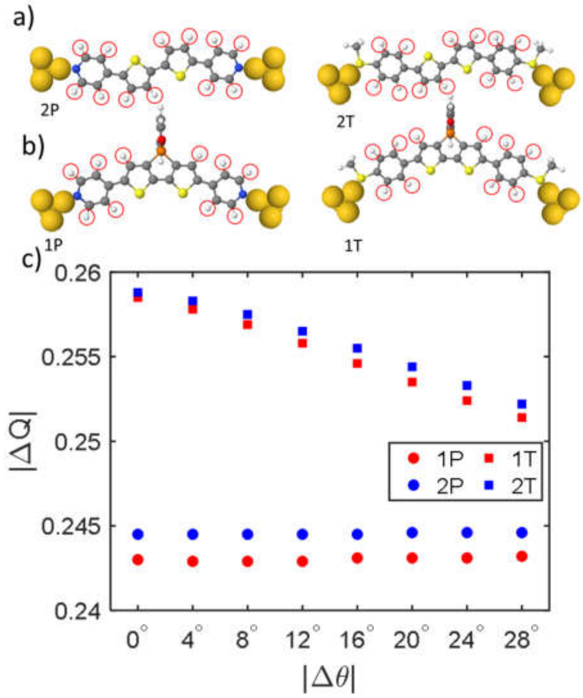

Figure S21. Charge density on hydrogen atoms. (a,b) Structure of the molecules (**1P**, **2P)** and (**1T**, **2T**) between Au electrodes for a range of configurations with different dihedral angles ($|\Delta\theta| = 4^o$). (c) Electron charge density on hydrogen atoms (red circle) in (**1P**, **2P)** and (**1T**, **2T**) as a function of the dihedral angle between the molecule and the Au electrodes.

Structure	HOMO-1	HOMO	Gap	LUMO	LUMO+1
	-5.37	-4.73	2.09	-2.64	-1.82
	-5.46	-4.85	1.97	-2.88	-1.89
	-4.47	-3.87	1.90	-1.97	-1.16
	-4.59	-3.99	1.74	-2.25	-1.25

Figure S22. Frontier orbital table for the gas-phase structures

4. Gas-Phase Calculations with B3LYP/6-31G*

In addition to the energy levels calculated with SIESTA, we present here the same calculations performed with Spartan22, using the B3LYP functional and the 6-31G* basis set.

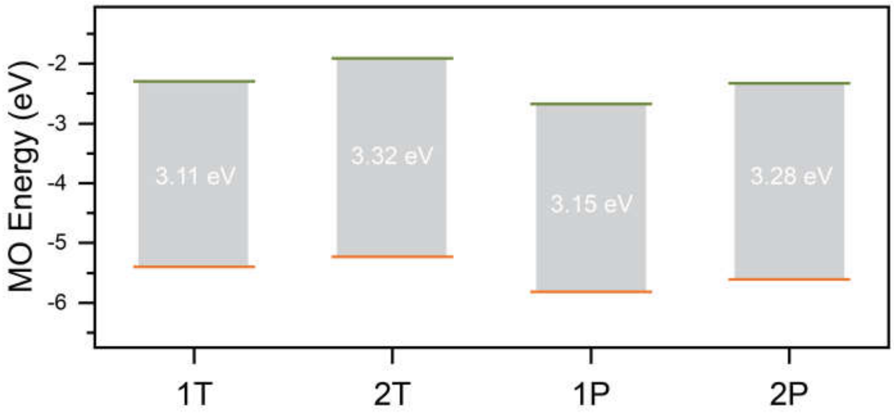

Figure S23. HOMO and LUMO energy levels (with bandgap showed as shaded region for the compounds used in this study. B3LYP/6-31G* level of theory. Wavefunction Spartan22.

Compared with the SIESTA results, the HOMO is consistently lower in energy with a resulting larger bandgap, but the overall trends remain the same. Values are reported below.

Table S2. Comparison between energy levels obtained with the two levels of theory used.

	**SIESTA (GGA/DZP)**				**SPARTAN (B3LYP/6-31G*)**			
	**HOMO**	**LUMO**	**E_g**	**ΔE_g**	**HOMO**	**LUMO**	**E_g**	**ΔE_g**
**1T**	-3.99	-2.25	1.74	0.26	-5.41	-2.30	3.11	0.21
**2T**	-3.87	-1.97	1.90		-5.23	-1.91	3.32	
**1P**	-4.85	-2.88	1.97	0.12	-5.82	-2.67	3.15	0.13
**2P**	-4.73	-2.64	2.09		-5.61	-2.33	3.28	

References

[1] P. Demay-Drouhard, T. Baumgartner, *J. Org. Chem.* **2020**, *85*, 14627–14633.

[2] A. Osadnik, A. Lützen, *Synthesis* **2014**, *46*, 2976–2982.

[3] N. Ferri, N. Algethami, A. Vezzoli, S. Sangtarash, M. McLaughlin, H. Sadeghi, C. J. Lambert, R. J. Nichols, S. J. Higgins, *Angew. Chem. Int. Ed.* **2019**, *58*, 16583–16589.

[4] C. Wu, X. Qiao, C. M. Robertson, S. J. Higgins, C. Cai, R. J. Nichols, A. Vezzoli, *Angew. Chem. Int. Ed.* **2020**, *59*, 12029–12034.

[5] E. Artacho, E. Anglada, O. Diéguez, J. D. Gale, A. García, J. Junquera, R. M. Martin, P. Ordejón, J. M. Pruneda, D. Sánchez-Portal, J. M. Soler, *J. Phys. Condens. Matter Inst. Phys. J.* **2008**, *20*, 064208.

[6] J. M. Soler, E. Artacho, J. D. Gale, A. García, J. Junquera, P. Ordejón, D. Sánchez-Portal, *J. Phys. Condens. Matter* **2002**, *14*, 2745–2779.

[7] J. Ferrer, C. J. Lambert, V. M. García-Suárez, D. Z. Manrique, D. Visontai, L. Oroszlany, R. Rodríguez-Ferradás, I. Grace, S. W. D. Bailey, K. Gillemot, H. Sadeghi, L. A. Algharagholy, *New J. Phys.* **2014**, *16*, 093029.
